# Questionable prospective effects of self-esteem on anxiety and academic self-efficacy: a simulated reanalysis and comment on Cao and Liu (2024)

**DOI:** 10.3389/fpsyg.2025.1572892

**Published:** 2025-06-17

**Authors:** Kimmo Sorjonen, Bo Melin

**Affiliations:** Karolinska Institutet (KI), Solna, Sweden

**Keywords:** academic self-efficacy, anxiety, cross-lagged panel model, self-esteem, simulation, spurious prospective effects, triangulation

## Abstract

**Objectives:**

The objective of the present simulated reanalysis was to scrutinize the conclusion by Cao and Liu that self-esteem can protect against anxiety and promote academic self-efficacy.

**Method:**

We simulated data to resemble the data used by Cao and Liu. We used triangulation and fitted complementary models to the simulated data.

**Results:**

We found contradicting decreasing and increasing effects of initial self-esteem on subsequent change in anxiety and academic self-efficacy. These divergent findings suggested that it is premature to assume a protective effect of self-esteem on anxiety and an enhancing effect on academic self-efficacy and the conclusions by Cao and Liu in this regard can be challenged.

**Discussion:**

It is important for researchers to be aware that correlations, including adjusted cross-lagged effects, do not prove causality in order not to overinterpret findings, something that appears to have happened to Cao and Liu. We recommend researchers to triangulate by fitting complementary models to their data in order to evaluate if observed effects may be due to true causal effects or if they appear to be spurious.

## Introduction

Self-esteem is often defined as our attitudes toward ourselves, where high and low self-esteem would mean a positive and a negative attitude, respectively (Kenrick et al. 2007). Self-esteem correlates with various important outcomes, e.g., quality of social relations (Harris and Orth, 2020), physical and mental health (including anxiety) (Sowislo and Orth, 2013; Trzesniewski et al. 2006), and academic self-efficacy (i.e., belief in one’s ability to achieve academic goals) (Di Giunta et al. 2013; Mao et al. 2020). However, correlations do not prove causality, e.g., because they may be due to the influence of some confounding factor (Reichenbach, 1971).

Cao and Liu (2024) analyzed longitudinal data (two waves of measurement, during third and fourth year of college, respectively) on self-esteem, anxiety, and academic self-efficacy in a sample of university students in China (approximately 21 years of age at the initial measurement, 48% females, *N* = 2,298) with cross-lagged panel models. Cao and Liu reported a statistically significant negative cross-lagged effect of initial self-esteem on subsequent anxiety when adjusting for initial anxiety and a statistically significant positive cross-lagged effect of initial self-esteem on subsequent academic self-efficacy when adjusting for initial academic self-efficacy. Cao and Liu interpreted their findings in causal terms, concluding, for example, that high self-esteem reduces the likelihood of experiencing anxiety and strengthens academic self-efficacy.

However, it is well established that adjusted cross-lagged effects may be spurious due to correlations with residuals and regression to the mean (Campbell and Kenny, 1999; Castro-Schilo and Grimm, 2018; Sorjonen et al. 2019). For example, there appears to be a negative correlation between self-esteem and anxiety (Cao and Liu, 2024), which could be due to a confounding impact by a third variable, e.g., general mental health or loneliness. Therefore, we should expect that among individuals with the same initial anxiety score, those with a higher self-esteem score have received a higher score on anxiety compared with their true anxiety, i.e., a more positive residual, while those with a lower self-esteem score have received a lower anxiety score compared with their true score, i.e., a more negative residual. However, residuals tend to regress towards a mean value of zero between measurements. Consequently, we should expect a more negative, but spurious, change in anxiety to a subsequent measurement among those with a higher initial self-esteem score compared with those with the same initial anxiety score but with a lower initial self-esteem score.

Would we, in agreement with Cao and Liu, interpret a negative cross-lagged effect of initial self-esteem on subsequent anxiety when adjusting for initial anxiety as a causal decreasing effect (not an interpretation we endorse), then, for consistency, could a negative effect of initial self-esteem on initial anxiety when adjusting for subsequent anxiety be interpreted as a causal increasing effect. This negative effect would indicate that low initial self-esteem had counteracted high initial anxiety and allowed individuals to reach the same subsequent level of anxiety as individuals with lower initial anxiety but higher initial self-esteem. Moreover, a positive effect of initial self-esteem on the subsequent - initial anxiety difference could be interpreted to suggest a causal increasing effect. Similarly, a positive effect of initial self-esteem on subsequent academic self-efficacy when adjusting for initial academic self-efficacy could, by some, be interpreted as a causal increasing effect, while a positive effect on initial self-efficacy when adjusting for subsequent self-efficacy, and a negative effect on the subsequent-initial self-efficacy difference, could be interpreted as causal decreasing effects. By assessing these predicted effects, we show how sensitivity of cross-lagged effects can be evaluated.

## Method

We estimated the effects outlined above in data simulated to resemble the empirical data used by Cao and Liu, with the same sample size and correlations between variables. We used simulated data as the empirical data were not available to us. The corresponding author of the study by Cao and Liu did not respond to our request for the data. It should be noted that both the standardized effect of X on Y_2_ when adjusting for Y_1_ (Equation 1, Cohen et al. 2003) and on the Y_2_-Y_1_ difference (Equation 2, Guilford, 1965) are functions of correlations between the variables. Consequently, these effects will be the same in data, empirical or simulated, with the same correlations between variables and simulated data can be used to estimate what the effects would have been in corresponding empirical data. For example, in the data used by Cao and Liu, the correlations between initial self-esteem and subsequent anxiety (*r_X, Y2_*), initial self-esteem and initial anxiety (*r_X, Y1_*), and initial and subsequent anxiety (*r_Y1, Y2_*) were −0.298, −0.378, and 0.530, respectively. Inserting these values into Equation 2 gives an expected effect of initial self-esteem on subsequent change in anxiety equal to *β* = 0.08. It is not conceivable that this effect would be negative in analyses of the empirical data, given this set of correlations.


(1) $$E(\beta_{X,Y2.Y1})=\frac{r_{X,Y2}-r_{X,Y1}r_{Y1,Y2}}{1-r_{X,Y1}^{2}}$$

(2) $$E(\beta_{X,Y2-Y1})=\frac{r_{X,Y2}-r_{X,Y1}}{\sqrt{2(1-r_{Y1,Y2})}}$$

Analyses and the simulation for the present study were conducted with R 4.4.0 statistical software (R Core Team, 2025) using the MASS package (Venables and Ripley, 2002). The analytic script, which also generates the simulated data, is available at the Open Science Framework at https://osf.io/hy2w3/ .

## Results

Initial self-esteem had a negative effect on subsequent anxiety when adjusting for initial anxiety (*β* = −0.114 [−0.151; −0.077], *p* < 0.001), a negative effect on initial anxiety when adjusting for subsequent anxiety (β = −0.242 [−0.277; −0.207], *p* < 0.001), and a positive effect on the subsequent-initial anxiety difference (*β* = 0.080 [0.040; 0.120], *p* < 0.001). In a taxonomy of treatment effects (Sorjonen et al. 2024), this combination of signs of effects suggests an increasing effect of initial self-esteem on subsequent change in anxiety, where a difference in anxiety between those with high and low initial self-esteem decreases from the initial to the subsequent measurement. However, we prefer the more cautious conclusion that the effects may have been spurious. Furthermore, initial self-esteem had a positive effect on subsequent academic self-efficacy when adjusting for initial academic self-efficacy (*β* = 0.130 [0.091; 0.169], *p* < 0.001), a positive effect on initial self-efficacy when adjusting for subsequent self-efficacy (β = 0.299 [0.264; 0.335], *p* < 0.001), and a negative effect on the subsequent-initial self-efficacy difference (β = −0.109 [−0.150; −0.068], *p* < 0.001). In a taxonomy of treatment effects (Sorjonen et al. 2024), this combination of signs of effects suggests a decreasing effect of initial self-esteem on subsequent change in self-efficacy, where a difference in self-efficacy between those with high and low initial self-esteem decreases from the initial to the subsequent measurement. However, we prefer, again, the more cautious conclusion that the effects may have been spurious.

## Discussion

The present findings suggest that the conclusions by Cao and Liu of a decreasing and an increasing effect of initial self-esteem on the subsequent change in anxiety and academic self-efficacy, respectively, were premature and may be challenged. It is important for researchers to bear in mind that correlations, including adjusted cross-lagged effects, do not prove causality in order not to overinterpret findings, something that appears to have happened to Cao and Liu. The present criticism is not exclusively directed at Cao and Liu but more broadly at causal conclusions (direct or indirect, e.g., in the form of policy recommendations) based on results from cross-lagged panel models. Effects in cross-lagged panel models usually do not prove causality any more than zero-order cross-sectional correlations do.

We recommend researchers to, as we did here, use triangulation by fitting complementary models to their data. If results from the models converge, conclusions of causality are corroborated (although never finally proven). If, on the other hand and as in the present case, findings diverge, conclusions of causality would appear premature. Diverging results would demonstrate the sensitivity and unreliability of cross-lagged effects. We would also recommend researchers to scrutinize other researchers’ questionable causal claims based on cross-lagged effects. Researchers scrutinizing each other’s work is an important part of science and promotes, directly or indirectly, the replicability and reproducibility of scientific findings. Unfortunately, researchers are often unwilling to share their data. However, conclusions based on results from analytic methods that are based on covariances, e.g., regression analyses and structural equation modeling (SEM), can be scrutinized by simulating data with the same sample size and covariances (or correlations, for standardized coefficients) between variables. The analytic script used for the present study (available at https://osf.io/hy2w3/) can easily be modified to simulate and analyze other data sets.

## Data Availability

Publicly available datasets were analyzed in this study. This data can be found here: The analytic script, which also generates the simulated data, is available at the Open Science Framework at https://osf.io/hy2w3/.
